# Podoplanin in cancer cells is experimentally able to attenuate prolymphangiogenic and lymphogenous metastatic potentials of lung squamoid cancer cells

**DOI:** 10.1186/1476-4598-9-287

**Published:** 2010-10-31

**Authors:** Hanako Suzuki, Mitsuho Onimaru, Yoshikazu Yonemitsu, Yoshihiko Maehara, Seiji Nakamura, Katsuo Sueishi

**Affiliations:** 1 Division of Pathophysiological and Experimental Pathology, Department of Pathology, Graduate School of Medical Sciences, Kyushu University, Fukuoka, Japan; 2 Department of Oral and Maxillofacial Surgery, Graduate School of Dental Sciences, Kyushu University, Fukuoka, Japan; 3 R&D Laboratory for Innovative Biotherapeutics Graduate School of Pharmaceutical Sciences, Kyushu University; 4 Department of Surgery and Science, Graduate School of Medical Sciences, Kyushu University, Fukuoka, Japan

## Abstract

**Background:**

Podoplanin, a mucin-like transmembrane glycoprotein, is reportedly expressed in a variety of malignant cells and is generally regarded as a factor for promoting tumor progression in conventional studies. By contrast, a clinicopathologically conflicting role for podoplanin, namely as a favorable prognostic factor for patients with lung/cervical squamous cell carcinoma (SCC), has recently been reported. Here, we investigated the role of podoplanin expressed in lung squamoid cancer cells (LSCCs) in experimental tumor progression.

**Results:**

Using EBC-1 cells, a lung SCC cell line without podoplanin expression and with lymphogenous metastatic potential, stable transformants with or without an exogenous human *podoplanin *gene were established and applied to a mouse tumor implantation model. *In vivo *examinations revealed that exogenous podoplanin had no influence on tumor growth, whereas it significantly restrained axillary lymph node metastasis associated with the suppression of lymphangiogenesis but not angiogenesis and with the downregulation of EBC-1-derived VEGF-C but not other lymphangiogenesis-related factor mRNAs in implanted tumor tissue. *In vitro *examinations to clarify the mechanisms underlying the *in vivo *phenomena revealed that exogenous podoplanin significantly suppressed the expression of VEGF-C mRNA and of the protein, and also increased the level of phosphorylated c-jun N terminal kinase (JNK) in EBC-1 cells. The former effect of exogenous podoplanin was impaired by treatment with either JNK inhibitor sp600125 or podoplanin-siRNA, and the latter effect was impaired by treatment with podoplanin-siRNA, suggesting that podoplanin was able to activate JNK, thereby downregulating VEGF-C gene expression in LSCCs (podoplanin-JNK-VEGF-C axis). Furthermore, supporting evidence in regard to the axis present in LSCCs was obtained from similar experiments using H157 cells, another lung SCC cell line expressing endogenous podoplanin.

**Conclusions:**

Our findings suggested that LSCC-associated podoplanin was functional and could attenuate the potential for lymph node metastasis, possibly based on the suppression of tumor lymphangiogenesis; thus, podoplanin in cancer cells may become a useful biomarker to measure the malignancy of lung SCC.

## Background

Lymphogenous and hematogenous metastases are major events in malignant tumor progression and important prognostic determinants of patients with cancer. Metastasis of cancer cells is a multi-step process, including malignant cell growth, cell detachment, invasion into adjacent tissue, blood or lymphatic permeation, entry into the blood or lymph flow, arrival at remote organ or lymph node, capillary arrest, extravasation, and proliferation within target organs [1]. Numerous factors expressed in tumor cells are implicated in the process of metastasis.

Lymph node status is one of the critical prognostic indicators in patients with malignant tumors, and tumor-associated lymphangiogenesis is thought to be a key step in promoting lymphogenous metastasis of malignant cells. A number of experimental and clinicopathological studies have supported the significance of lymphangiogenesis in tumor progression, including non-small cell lung carcinoma [2-5]. Tumor lymphangiogenesis is regulated by lymphangiogenesis-related growth factors expressed in malignant cells and cognate receptors expressed in host lymphatic vessels [6-17]. Especially, paracrine interaction between vascular endothelial growth factors (VEGF)-C and -D, and their cognate receptor, VEGF receptor-3, plays a central role in tumor lymphangiogenesis in a variety of malignancies [4]. In many cases, a high expression level of VEGF-C in malignant tumor cells correlates with increased density of peritumoral lymphatic vessels, increased incidence of lymph node metastasis, and poor prognosis [17].

Podoplanin is a mucin-like transmembrane glycoprotein [18]. Since its expression is completely restricted in lymphatic endothelial cells in the vascular system, it is now available as a useful marker to distinguish lymphatic vessels immunohistochemically from blood vessels [19,20]. Podoplanin is also expressed in a variety of non-neoplastic cells such as podocytes and alveolar type-I cells [18-23]. According to a recent gene targeting study, podoplanin^-/- ^mice showed systemic edema due to aplastic lymphatic vessels during fetal development, and neonatal death due to respiratory failure [24,25]. These findings are suggestive of the multi-physiological functioning of podoplanin in a cell-type-specific manner.

Recently, podoplanin has been reported to be expressed in a variety of malignant tumor cells, such as squamous cell carcinoma, methothelioma, and germ cell tumors [22,26], and evidence suggesting the involvement of podoplanin in malignant potential from various studies has accumulated: 1) Podoplanin can alter cell morphology and motility, by which tumor invasive/migratory activity is promoted [27,28]; 2) Podoplanin can induce the epithelial-mesenchymal transition [29]; and 3) Podoplanin can induce platelet activation/aggregation mediated by its platelet aggregation-stimulating (PLAG) domain, resulting in a greater ability to achieve hematogenous metastasis of circulating tumor cells [30-32]. Together, previous *in vitro *and *in vivo *experimental studies have suggested that podoplanin is an enhancer that promotes tumor progression

The role of podoplanin in tumor cells, however, seems to be controversial in recent clinicopathological studies of human cancers. For example, Yuan *et al*. and Chuang *et al*. demonstrated that a higher expression level of podoplanin in cancer cells significantly correlated with poor prognosis and a higher incidence of lymph node metastasis in head and neck SCC [33,34]. Conversely, current several reports demonstrate that a lower expression level of podoplanin in cancer cells significantly correlated with a poor prognosis and a higher incidence of lymph node metastasis in both lung and cervical SCCs [35-37]. Hitherto-existing evidence relating to podoplanin functions can not explain the mechanism underlying the tumor suppression. The inconsistent and elusive results of these traditional experimental and clinicopathological studies may suggest that podoplanin exerts context-dependent multi-functions in different organ environments and/or different malignant cells, and that it may act as an enhancer in some cases and a suppressor in others in tumor progression.

Here, we investigated the role of cancer cell-associated podoplanin in the progression of lung SCC using an animal model, and found novel functions of podoplanin as a suppressor for cancer progression.

## Results

### Podoplanin does not affect activities of cell growth and migration *in vitro*

**Figure 1:**
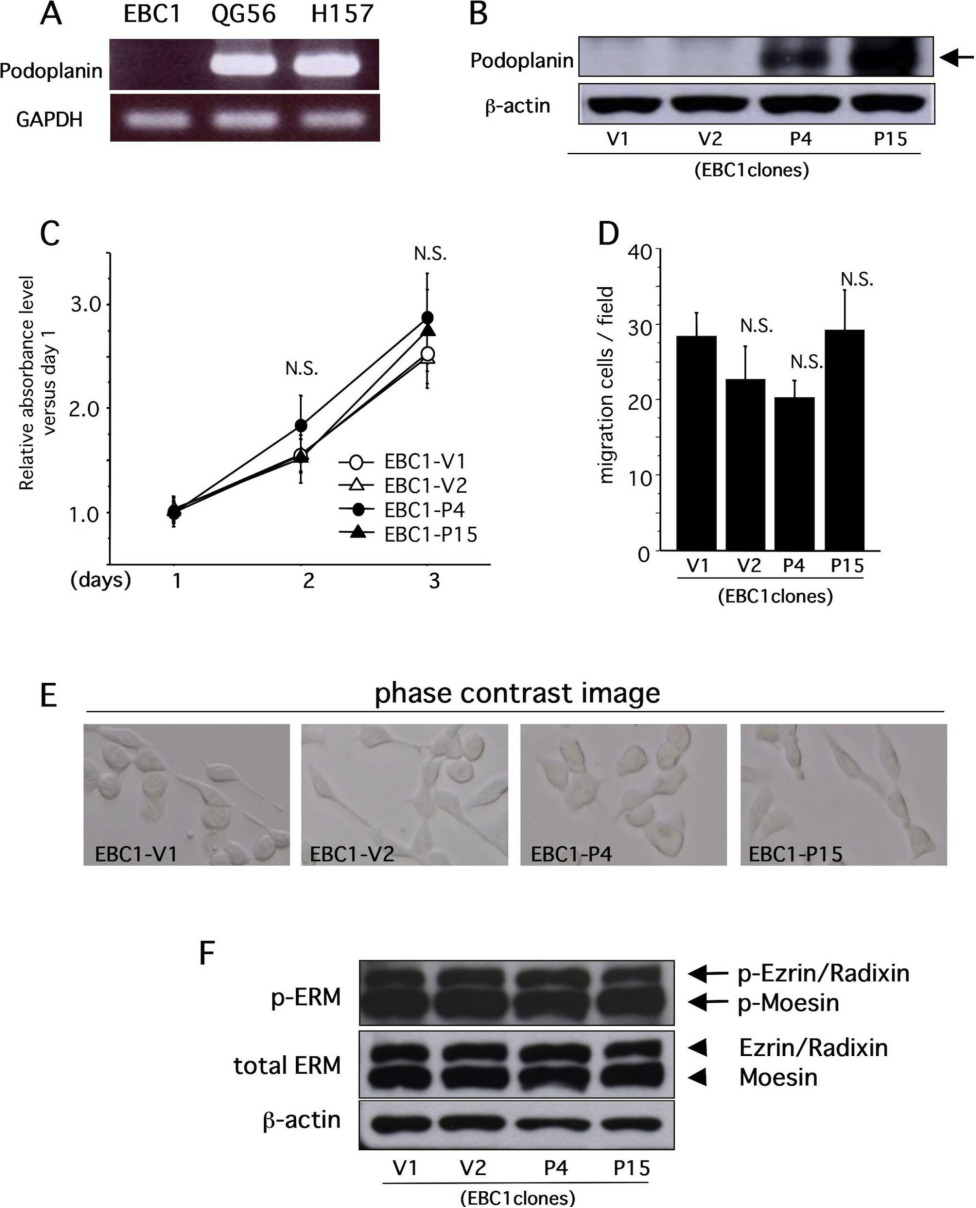
**Effect of podoplanin expression in tumor cells on cellular function *in vitro***. A) RT-PCR for human podoplanin and GAPDH was performed with cDNA from LSCC cell lines QG56, H157, and EBC-1 as described in Methods. B) Cell lysates from cultured stable clones indicated were subjected to Western blot analysis for human podoplanin and subsequently to re-probing for β-actin. Exogenous podoplanin expression was confirmed (V1 and V2, control stable clones 1 and 2 with empty vector; P4 and P15, stable clones 4 and 15 with podoplanin cDNA-inserted vector) C) The growth activity of each established clone indicated was examined as described in Methods. (N.S., no significant change versus V1). D) Migratory activities of each established clone were examined as described in Methods. No significant change in migratory activity was evident among EBC1 clones (N.S. versus V1). E) The phase contrast image of each cultured clone is shown. F) Cell lysates from the cultured stable clones indicated were subjected to Western blot analysis for phosphorylated ERM (p-ERM) molecules (p-Ezrin/Radixin and p-Moesin, arrows) and subsequently to re-probing for total ERM (Ezrin/Radixin and Moesin, arrowheads) and β-actin.

First, we examined the expression level of podoplanin in a variety of lung SCC cell lines. RT-PCR revealed that only EBC-1 cells showed no podoplanin expression (Figure 1A). This finding was confirmed by real-time PCR (data not shown). In addition, 21 days after cutaneous inoculation of EBC-1 cells in the dorsal area of BALB/c nu/nu mice, approximate 65% mice showed axillary lymph node metastasis, suggesting that EBC-1 cells possessed high lymphogenous metastatic potential and were available for validating the effect of podoplanin in lymphogenous metastasis. Then, EBC-1 cell-derived stable transformants with or without cDNA of human *podoplanin *were established. Eventually, 15 single clones were established, and the two of these (EBC1-Ps: EBC1-P4 and EBC1-P15) that had the highest expression of podoplanin mRNA were adopted (data not shown). On the contrary, 3 single clones with empty vectors were simultaneously established and two clones (EBC-Vs: EBC1-V1 and EBC1-V2) were randomly adopted as controls. Protein expression of exogenous podoplanin was confirmed by Western blot analysis (Figure 1B). These clones were used in a series of experiments in this study. Using these stable transformants, we examined the effects of podoplanin on EBC-1 migration and proliferation activities. As a result, podoplanin had no influence on the proliferation and migration of EBC-1 cells *in vitro *as evidenced by Figures 1C and 1D. Regarding the negative data obtained from the migration assay, we performed additional experiments to indicate a positive control, which revealed that stable overexpression of exogenous podoplanin could promote the migration activity in SAS cells, an oral SCC cell line (additional file 1). This finding strongly suggested that podoplanin had no influence on the migration activity of LSCCs *in vitro*. Moreover, no apparent changes in cell morphology, such as filopodia-like formation, could be observed in EBC1-Ps (Figure 1E). The morphological appearances were supported by the results of Western blot analysis, which demonstrated that the phosphorylated levels of ERM molecules (ezrin/radixin/moesin), which are implicated in cellular actin cytoskeleton rearrangement, were not affected by podoplanin overexpression in EBC-1 cells (Figure 1F). These findings except for the proliferation data are not consistent with those in previous reports [27], in which podoplanin could lead to enhanced migration activity and to inducement of filopodia-like formation based on remodeling of actin cytoskeleton.

### Podoplanin attenuates lymphogenous metastatic potential *in vivo*

**Figure 2:**
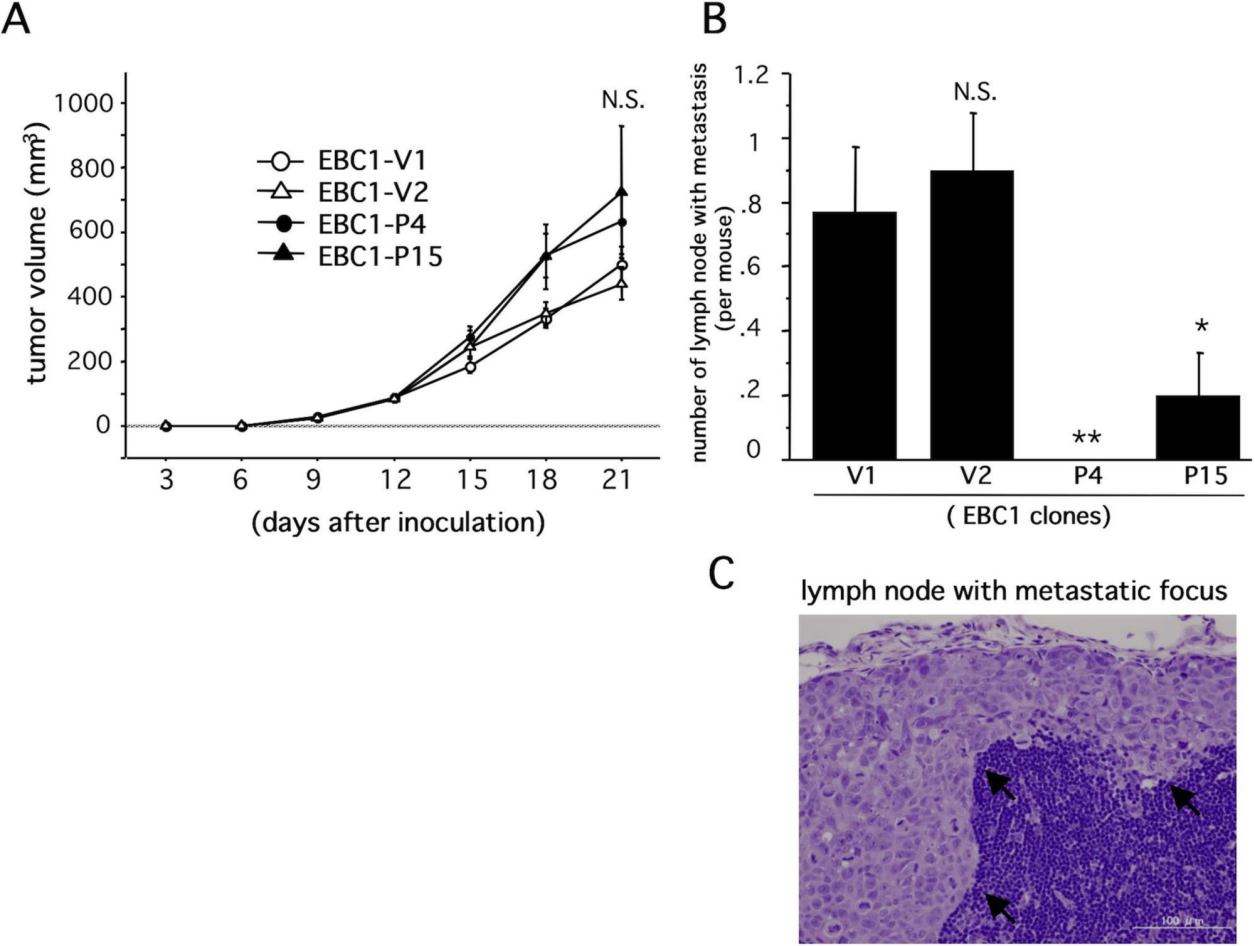
**Suppressive effect of tumor cell-associated podoplanin on lymph node metastasis in our animal model**. A) The time-dependent tumor volume derived from each established clone in a tumor implantation model was measured and evaluated as described in Methods. (N.S. versus V1). B) The incidence of lymph node metastasis of each group was evaluated as described in Methods (**p < 0.001 versus V1, *p < 0.05 versus V1, N.S. versus V1). C) Representative metastatic foci in axillary lymph nodes of mice with implanted tumors are indicated (arrows).

Next, we examined the effect of podoplanin on the tumor growth and lymphogenous metastatic potential using a tumor implantation model as described in Methods. As a result, the growth activity of EBC1-P-derived tumors showed no significant difference compared to that of EBC1-V-derived tumors (Figure 2A). By contrast, EBC1-P cell-implanted mice exhibited a significantly and markedly reduced incidence of axillary lymph node metastasis compared to EBC1-V1 cell-implanted mice (Figure 2B). A representative lymph node specimen is shown in Figure 2C. These results suggested that podoplanin could help to reduce the potential of lymphogenous metastasis of LSCCs.

### Podoplanin inhibits tumor-associated lymphangiogenesis but not angiogenesis

**Figure 3:**
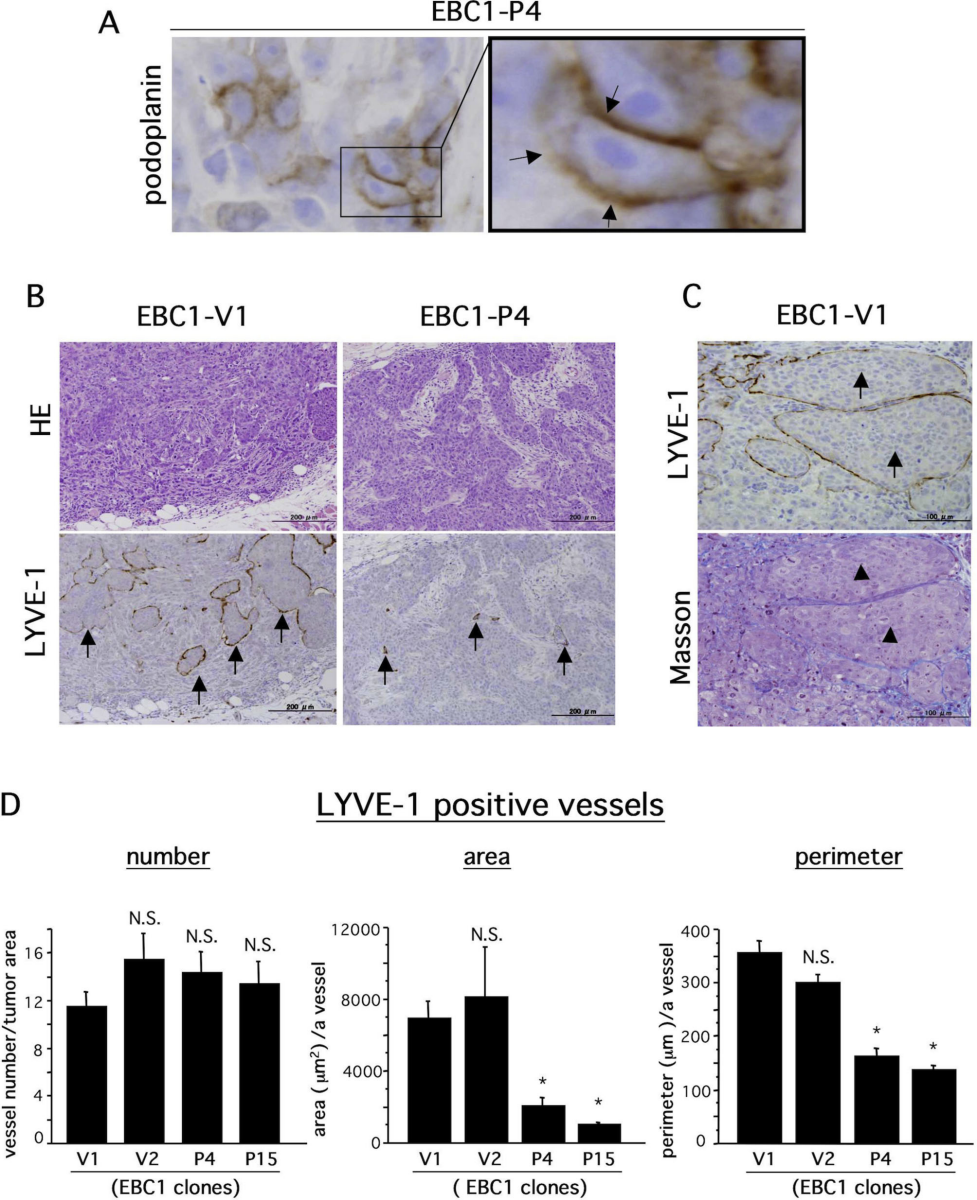
**Suppressive effect of tumor cell-associated podoplanin on tumor lymphangiogenesis**. A) Immunohistochemical staining for human podoplanin with EBC1-P4-derived tumor sections. Representative results are indicated. Right photograph is a magnification of the box shown in the left photograph. Podoplanin expression was observed at the cell surface (arrows). B) Implanted tumor tissues were subjected to HE staining and immunohistochemical staining for mouse LYVE-1. Representative photographs (for EBC1-V1 and EBC1-P4) are indicated. LYVE-1-positive signals (arrows) were observed. C) Serially sectioned tumor tissues derived from EBC1-V1 were subjected to immunohistochemical staining for mouse LYVE-1 (upper photograph) and to Masson staining (lower photograph). Tumor cell nests surrounded by podoplanin-positive lines (arrows) showed a negative reaction to Masson staining (arrowheads). D) The area, the perimeter, and the number of LYVE-1-positive lymphatic vessels were measured and evaluated as described in Methods (n = 3 each, *p < 0.001 versus V1).

**Figure 4:**
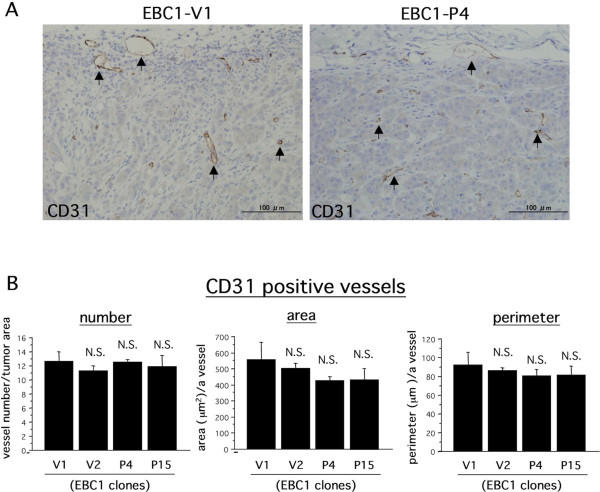
**No significant effect of tumor cell-associated podolanin on tumor angiogenesis**. A) Implanted tumor tissues were subjected to immunohistochemical staining for mouse CD31 using serial sections as described in Methods. Representative photographs (for EBC1-V1 and EBC1-P4) are indicated. CD31-positive vessels were observed (arrows). B) The area, the perimeter, and the number of CD31-positive vessels were measured and evaluated as described in Methods (N.S. versus V1).

We hypothesized that the reduced potential of lymphogenous metastasis in EBC1-Ps was due to podoplanin-mediated inhibition of tumor-associated lymphangiogenesis. To validate our hypothesis, we performed an immunohistochemical study for blood and lymphatic vessels, using sections of implanted tumor tissues. First, we confirmed exogenous podoplanin expression in tumor tissue. As shown in Figure 3A, human podoplanin expression was immunohistochemically detected at the tumor cell surface in EBC1-P4-derived tumor tissue. Next, we evaluated tumor-associated lymphangiogenesis as described in Methods. Representative results of immunohistochemical staining for LYVE-1 are shown in Figure 3B. As evidenced by Figure 3B, many various-sized tumor cell nests circumscribed with the LYVE-1-positive brown signal were observed in EBC1-V1-derived tumor tissue (Figure 3B, EBC1-V1/LYVE-1, arrows). Since we had never seen such a LYVE-1-positive staining pattern, we used the alternative lymphatic endothelial marker podoplanin to perform an additional immunohistochemical examination to make sure the LYVE-1-positive brown signal was truly derived from lymphatic endothelial cells. As a result, we confirmed that mouse podoplanin had a staining pattern similar to that of LYVE-1, indicating that the tumor cell nests were circumscribed with lymphatic endothelial cells (additional file 2). In addition, to determine whether or not the tumor cell nests circumscribed with LYVE-1-positive lymphatic endothelial cells are in dilated lymphatic vessels, we performed Masson staining to visualize tumor-associated fibrous stroma. As shown in Figure 3C, no stromal component was revealed in tumor cell nests circumscribed with LIVE-1-positive endothelial cells with Masson staining, whereas the surrounding tumor tissue apparently showed Masson-staining-positive fibrous stroma, indicating that tumor cell nests were present in the dilated lymphatic vessels. This immunohistochemical evidence suggested that EBC-1 cells strongly possessed the ability to induce lymphangiogenesis and permeate lymphatic vessels, frequently with the result of lymphogenous metastasis. In accord with the lymphangiogenic character of EBC1 cell-derived tumor, we evaluated lymphangiogenic states, using several indexes including the vessel area and perimeter. The area and perimeter of LYVE-1-positive lymphatic vessels in viable tumor tissue were significantly lower in EBC1-P-derived tumors than in EBC1-V-derived tumors (Figure 3D). Surprisingly, the number of lymphatic vessels showed no significant difference (Figure 3D). We further evaluated blood angiogenic states. Blood vessels were immunohistochemically identified as CD31-positive vessels (Figure 4A), and were evaluated with the same indexes as in the case of lymphangiogenesis. The results showed that blood vessels were not significantly different for any indexes (Figure 4B). These histological findings suggest that podoplanin in LSCCs contributed to the inhibition of tumor-associated lymphangiogenesis.

### Podoplanin suppresses endogenous VEGF-C but not other lymphangiogenesis-related growth factor gene expressions *in vivo *and *in vitro*

**Figure 5:**
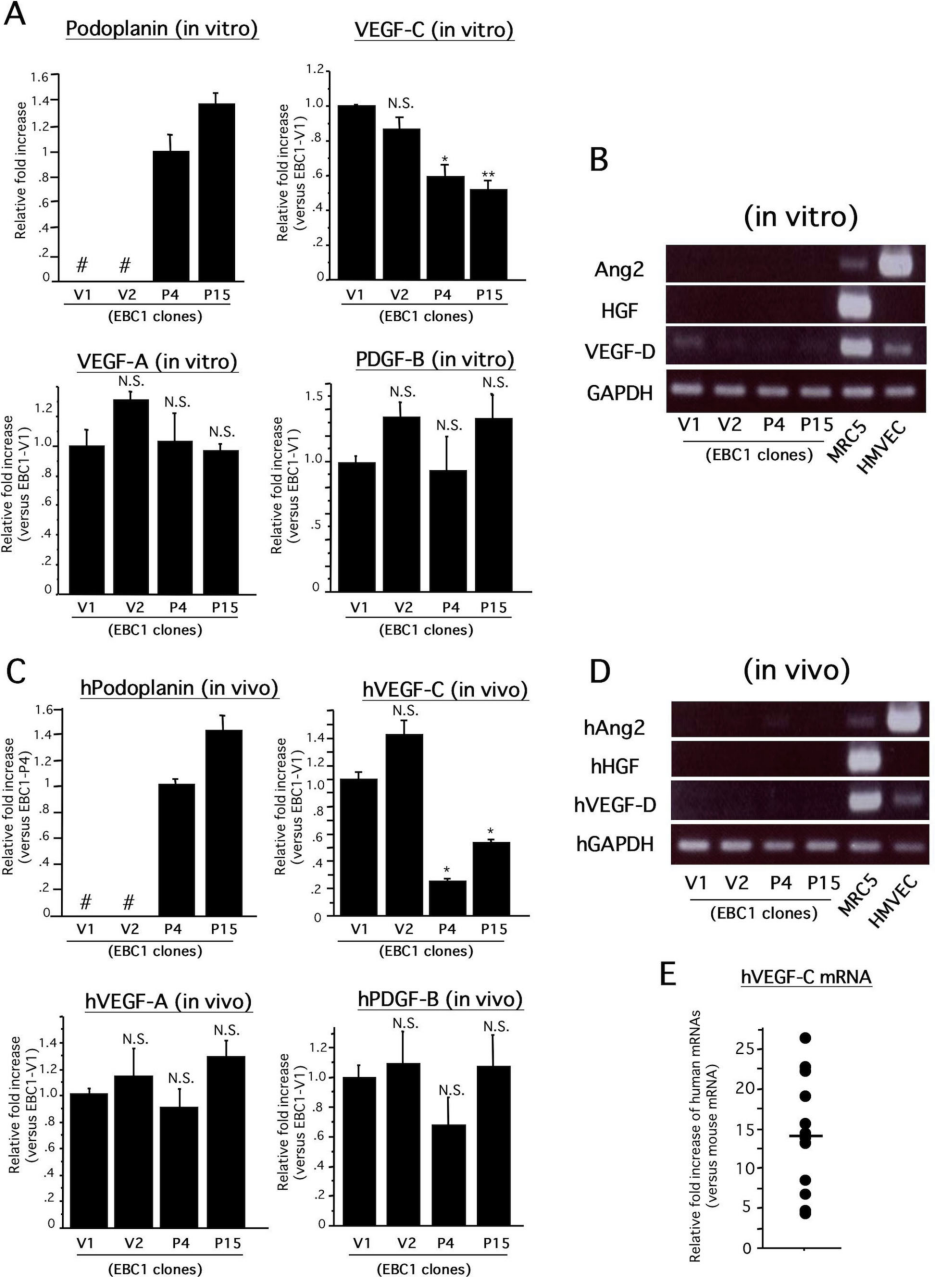
**Comparison of expression levels of lymphangiogenesis-related factors among the clones**. A) Each clone cultured was subjected to real-time RT-PCR for indicated factors. (n = 3 each, *p < 0.01 versus V1, **p < 0.001 versus V1, N.S. versus V1, # undetectable). B) Each clone cultured was subjected to RT-PCR for indicated growth factors. PCR primer sets are listed in additional file 4. cDNA templates from HMVEC and MRC5 were simultaneously amplified as positive controls. C) Each clone-derived tumor tissue was subjected to real-time RT-PCR for indicated human (h) growth factors using the human gene-specific primer sets listed in the additional file 4. (n = 3, *p < 0.001 versus V1, N.S. versus V1). D) Each clone-derived tumor tissue was subjected to RT-PCR for indicated human (h) growth factors using the human gene-specific primer sets listed in the additional file 3. cDNA templates from HMVEC and MRC5 were simultaneously amplified as positive controls. E) Tumor tissues derived from established clones (EBC1-V1, -V2, -P4, and -P15; n = 3 each) were subjected to real-time RT-PCR for human or mouse VEGF-C using the species-specific primer sets listed in the additional file 4. Plasmid templates inserting a part of human or mouse VEGF-C gene were prepared for standard curves as described in Methods. From the obtained data, the number of each template in the tumor-derived cDNA library was determined. The human VEGF-C mRNA level was expressed as a relative fold increase compared to that of the mouse VEGF-C level. Horizontal bar means average score.

To clarify the mechanisms by which tumor-associated lymphangiogenesis was suppressed in EBC1-P-derived tumor tissues, we examined the expression levels of proven pro-lymphangiogenic growth factors [7-9,13,15,16]. In our previous study, we reported that VEGF-A and VEGF-C mRNAs are apparently detectable by RT-PCR in wild-type EBC-1 cells. By contrast, the mRNA expressions of PDGF-B, VEGF-D, Angiopoietin (Ang)-2 and HGF were weak or undetectable [38]. Therefore, we quantitatively examined the expression levels of VEGF-A and VEGF-C in each clone *in vitro*. Real-time RT-PCR revealed that the expression levels of VEGF-C mRNA but not of VEGF-A were significantly reduced in EBC1-Ps compared to those in EBC1-Vs under culture conditions (Figure 5A). In addition, PDGF-B mRNA expression was weak but quantitatively able to be detected by real-time RT-PCR and showed no significant change among the clones (Figure 5A). Consistent with the gene expression pattern, the ELISA assay revealed that VEGF-C but not VEGF-A content in culture media was significantly reduced in EBC1-Ps, and that PDGF-BB content was undetectable (additional file 3). The expression levels of VEGF-D, Ang-2 and HGF were extremely weak or undetectable in each clone as well as in wild-type EBC-1 cells (Figure 5B). Next, we examined the EBC1 cell-derived mRNA levels of human VEGF-A, VEGF-C and PDGF-B in implanted tumor tissues as described in Methods. Real-time RT-PCR (Figure 5C) and RT-PCR (Figure 5D) using human specific primer sets revealed similar results compared to those from the *in vitro *experiments. We further examined the gene expression level of EBC1-derived human VEGF-C compared to that of mouse VEGF-C in implanted tumor tissue. As shown in Figure 5E, the human mRNA level was dramatically higher than the mouse mRNA level, suggesting that the level of EBC1-derived VEGF-C is predominant compared to that of host VEGF-C in the implanted tumor tissues. These findings may be insufficient to determine the significance of VEGF-C for inducing tumor lymphangiogenesis in our animal model; however, VEGF-C expression in EBC-1 cells is thought to be a key factor to induce tumor-associated lymphangiogenesis, and podoplanin-mediated downregulation of the VEGF-C gene might be a possible mechanism underlying the different levels of lymphangiogenic activity between EBC1-Vs and EBC1-Ps.

### Podoplanin-induced VEGF-C downregulation is mediated by an increase in the level of activated JNK in LSCCs

**Figure 6:**
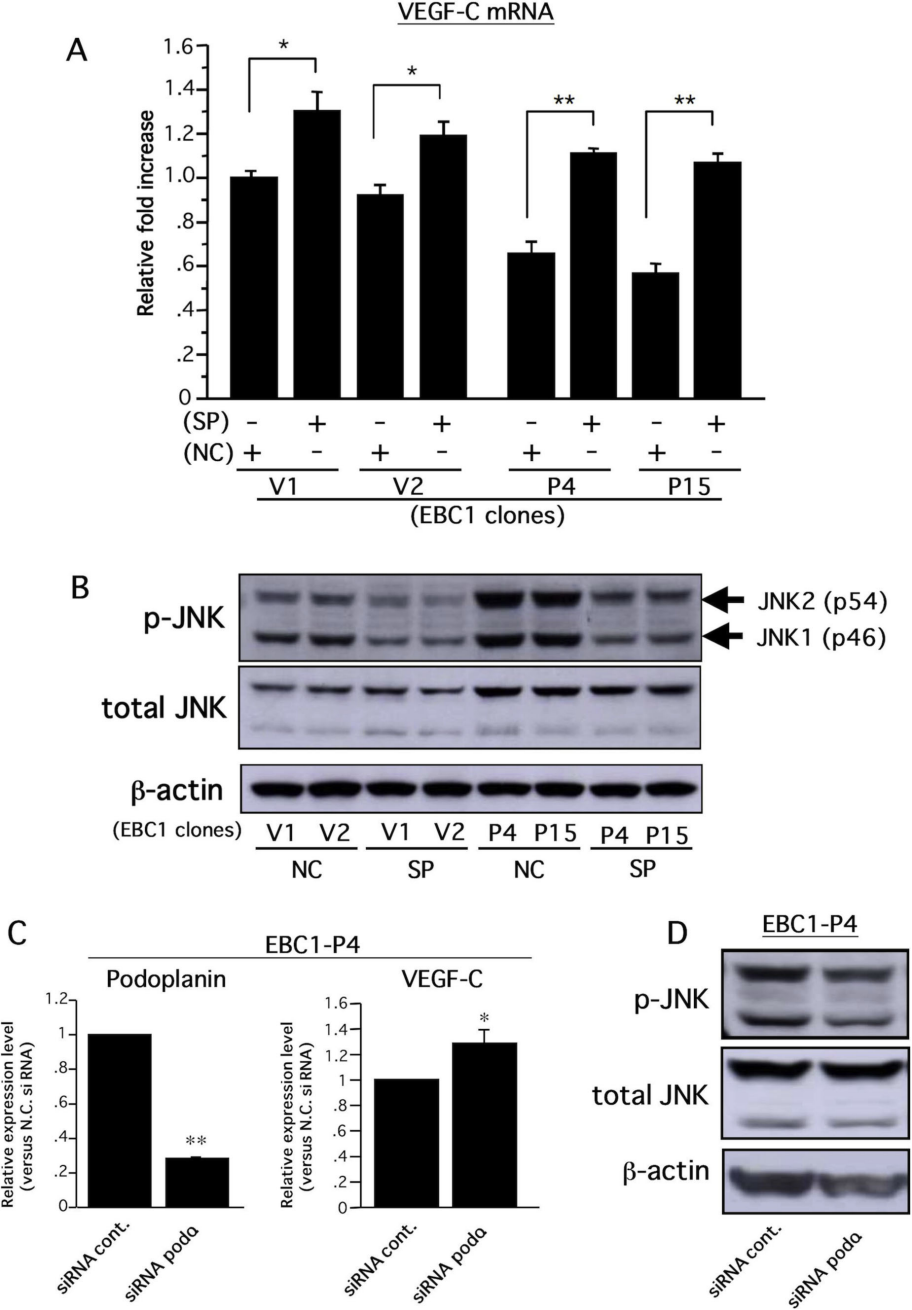
**Podoplanin-dependent VEGF-C downregulation is required for JNK activity**. A) Following 24-hour cultivation of each clone under serum-free conditions, the medium was replaced with fresh serum-free medium containing JNK inhibitor, sp600125 (SP, 20 μM), or a negative control regent (NC, 20 μM) and incubated for 6 hours. Treated cells were harvested, and subjected to real-time RT-PCR for VEGF-C and GAPDH. The VEGF-C mRNA level normalized with each GAPDH level was expressed as a relative fold increase compared to that in EBC1-V1 (n = 3 each, *p < 0.05, **p < 0.005). B) After the same treatment as described in A, the cells were harvested and subjected to Western blot analysis for phospho-JNK and subsequently to re-probing for total JNK and then to β-actin. Two isoforms of JNK with different molecular weights, JNK1 (p46) and JNK2 (p54) were detected (arrows). C) SiRNA for human podoplanin and negative control oligonucleotides (siRNA podo. and siRNA cont., respectively) were transfected to EBC1-P4 cells as described in Methods. Twenty-four hours after transfection, the medium was replaced with serum-free fresh medium. Twenty-four hours later, cells were harvested and subjected to real-time RT-PCR for human podoplanin, VEGF-C, and GAPDH. Podoplanin and VEGF-C mRNA levels were normalized with the GAPDH level and expressed as relative fold increases compared to those of the control group (n = 3 each, *p < 0.05, **p < 0.0001). D) Following similar treatments of siRNA to those described in C, the medium was replaced with serum-free fresh medium. Six hours later, cells were harvested and subjected to Western blot analysis for phosphorylated JNK and subsequent re-probing for total JNK and β-actin.

**Figure 7:**
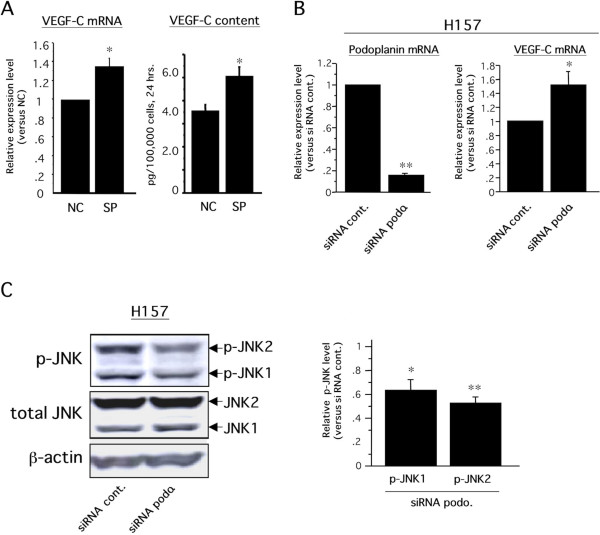
**Podoplanin-JNK-VEGF-C axis in H157 cells**. A) Experiments similar to those described in Figure 6A were performed using H157. VEGF-C mRNA level (left graph) and VEGF-C content (right graph) in the culture media were assessed by real-time RT-PCR and ELISA, respectively, as described in Methods. For the ELISA, culture media were harvested twenty-four hours after treatment with inhibitor (n = 3 each, *p < 0.05). B) Similar experiments described in Figure 6C were performed using H157 (n = 3 each, *p < 0.05, **p < 0.0001). C) Similar experiments described in Figure 6D were performed using H157 cells. Each band intensity was measured as described in Methods, and the level of phosphorylated JNK1 (p-JNK1) or p-JNK2 was normalized with each total JNK level, and expressed as a relative fold increase compared to the control. (n = 3 each, *p < 0.05 versus siRNA cont., **p < 0.01 versus siRNA cont.).

To identify the critical intracellular signal transductions by which podoplanin inhibited VEGF-C expression in EBC-1 cells, we examined the effects of intracellular signal inhibitors related to podoplanin-mediated downstream signals on VEGF-C expression. Recent studies have demonstrated that podoplanin is involved in intracellular signals of the Rho family composed of RhoA, Rac-1, and cdc42 [27,29,39]. Therefore, we focused on the relationships among podoplanin, Rho family-mediated downstream signaling molecules, ROCK/Rho kinase and JNK, and VEGF-C. Real-time RT-PCR revealed that the VEGF-C gene expression level in EBC1-Ps treated with sp600125, a JNK inhibitor, was significantly improved, nearly to the level found in EBC1-Vs (Figure 6A), whereas no change in the VEGF-C expression was observed in EBC1-Ps treated with the ROCK inhibitor Y-27632 (data not shown). Western blot analysis revealed that EBC1-Ps had higher JNK phosphorylation levels than EBC1-Vs, and sp600125-treated EBC1-Ps showed similar levels of phosphorylated JNK as in EBC1-Vs (Figure 6B). To further validate whether the increased activation of JNK-mediated downregulation of VEGF-C is dependent on podoplanin, the levels of phosphorylated JNK and VEGF-C mRNA were examined using siRNA methods. As a result, significant upregulation of VEGF-C mRNA (Figure 6C) and a decreased level of phosphorylated JNK (Figure 6D) were induced in EBC1-P4 cells treated with siRNA-podoplanin. Taken together, these findings suggested that the podoplanin-mediated increase in the level of activated JNK was a downregulation signal for the VEGF-C gene in EBC-1 cells. To enhance the credibility and universality of the podoplanin-JNK-VEGF-C axis in LSCCs, we further performed *in vitro *examinations, using H157, a lung SCC cell line with podoplanin expression (Figure 1A). Consistent with the results shown in Figure 6, VEGF-C gene expression and protein secretion were significantly upregulated in H157 cells treated with the JNK inhibitor sp600125 (Figure 7A). In addition, significant upregulation of VEGF-C mRNA (Figure 7B) and a decreased level of phosphorylated JNK (Figure 7C) were also induced in H157 cells treated with siRNA-podoplanin. On the contrary, we previously reported the intracellular signaling pathways, p42/44 MAPK and p38 MAPK, by which the VEGF-C gene is stimulated in the oral SCC cell line [38]. Although a 20 μM concentration of sp600125 reportedly had no influence on p42/44 MAPK and p38 MAPK activities in cultured cells [40], we confirmed the effect of sp600125 on the phosphorylation levels of p42/44 MAPK and p38 MAPK in EBC-1 and H157 cells. Consistent with the previous report [40], the phosphorylation levels of these molecules were hardly affected by sp600125 treatment at a 20 μM concentration in these cells (data not shown). Taken together, these findings suggest that JNK is a key pathway for inducing podoplanin-mediated downregulation of the VEGF-C gene in LSCCs.

## Discussion

Using stable lung SCC cell line-derived transformants exogenously expressing podoplanin, we herein found direct evidence suggesting the role of podoplanin in experimental tumor progression. The body of our findings is that podoplanin in LSCCs can induce VEGF-C downregulation via the JNK signaling pathway(s), and can impair tumor-associated lymphangiogenesis and lymphogenous metastasis

Current immunohistochemical studies have revealed the expression of podoplanin in a variety of malignant cells. In the case of SCCs, podoplanin is mainly expressed in peripheral cancer cells in solid nests [22,34,36]. A series of these past studies relevant to tumor cell-associated podoplanin suggest its role in promoting cancer progression, especially in enhancing the potential of cancer cell invasion, and this hypothesis has been supported by several past studies [41]. Indeed, previous reports have shown experimental evidence of interesting and important cytophysiological and cytochemical phenomena mediated by podoplanin. Notably, as evidenced by previous studies, podoplanin does not exert the same function in all cell types. For example, although podoplanin can induce RhoA activation and an epithelial-mesenchymal transition (EMT) in MDCK cells [29], it attenuated RhoA activity and could not induce EMT in a breast carcinoma cell line [27], suggesting that podoplanin exerts cell type-specific functions. Therefore, we are concerned about the interpretation of podoplanin's functions in several past studies. In fact, the function-related data were often from experiments using malignant cell lines that lacked podoplanin expression in actual human lesions. For example, since it has been immunohistochemically reported that almost all adenocarcinomas cells, including lung cancer, hardly express podoplanin [22,23], the evidence from experimental studies on podoplanin functions using such cell lines/animal models may be rather different from the pathophysiological roles it plays in human malignancies. Consequently, experimental studies should be designed with human cell lines that actually express podoplanin in human malignancies in order to understand its true role in human pathology. In the present study with lung SCC cell line EBC-1, several different results relating to podoplanin functions were observed compared to those reported in past studies: 1) podoplanin did not promote cell migration, and 2) podoplanin-mediated morphological change, such as filopodia-like formation, was not observed. These findings should be interpreted as relating only to LSCC-associated podoplanin function.

We have previously reported that VEGF-C expression is positively regulated by p42/44 MAPK, protein kinase C (PKC) or p38 MAPK in oral squamoid cancer cells [38]. In contrast, a negative regulatory mechanism has not been reported in any past studies. Here, we found the critical intracellular signaling pathway, JNK, for the negative regulation of VEGF-C gene. A decreased level of phosphorylated JNK but not of total JNK was induced by siRNA-mediated podoplanin knockdown (Figures 6D and 7C), suggesting that podoplanin could induce JNK phosphorylation. By contrast, as shown in Figure 6B, not only the phosphorylated JNK but also the total JNK levels were apparently increased in stable transformants exogenously expressing podoplanin. These phenomena may be due to the podoplanin-dependent phenotypic change of EBC-1 cells. As described above, podoplanin has the potential to induce EMT [29], so forced and long-term expression of podoplanin in our experimental conditions may cause several phenotypic changes of EBC-1 cells, resulting in the increased level of total JNK. Taken together, podoplanin-dependent downregulation of VEGF-C is mediated by the direct and/or indirect increase of active JNK level in LSCCs.

EBC1-induced lymphatic vessels in the implanted tumor were markedly dilated, frequently resulting in lymph node metastasis. In this model, podoplanin narrowed lymphatic vessels in luminal size. Since VEGF-C is reportedly an inducer not only in proliferation/migration of lymphatic endothelial cells but also in the enlargement of lymphatic vessels [42], podoplanin-mediated dowregulation of VEGF-C is a consistent mechanism for the decreased area and perimeter of lymphatic vessels. By contrast, the reason why podoplanin-mediated dowregulation of VEGF-C has no influence on the lymphatic vessel density remains unclear. Inoculated EBC-1 cells might be able to permeate the existing lymphatic vessels in an early phase of tumor growth and gradually proliferate in solid nests in lymphatic vessels. As a consequence, intralymphatic EBC-1 cell-derived VEGF-C acts on the free surface of lymphatic endothelial cells, thereby impairing concentration gradient-dependent vessel migration/sprouting but not affecting vessel proliferation/enlargement in our animal model.

Considering that a positive correlation between tumor VEGF-C and tumor lymphangiogenesis/lymphogenous metastasis has been reported in numerous malignancies including non-small cell lung carcinoma [5], we herein found a novel molecular mechanism, the podoplanin-JNK-VEGF-C axis, by which podoplanin impaired lymphogenous metastasis in our animal model. We could not, however, demonstrate any direct evidence suggesting a linkage between the podoplanin-JNK-VEGF-C axis and the podoplanin-dependent impairment of lymphangiogenesis/lymphogenous metastasis. Therefore, further examination is necessary to clarify the mechanism by which podoplanin suppresses lymph node metastasis, including the possibility that podoplanin is critical in other lymphangiogenesis-independent steps to achieve lymph node metastasis.

## Conclusions

All together, the findings obtained from our animal model in the present study were experimentally able to support recent clinicopathological evidence suggesting that the expression of podoplanin in cancer cells is a favorable prognostic marker of patients with lung SCC. Moreover, the podoplanin-mediated downregulation of the VEGF-C gene via JNK (the podoplanin-JNK-VEGF-C axis) was newly found as one of the possible underlying mechanisms. Therefore, podoplanin may be a useful prognostic biomarker to determine the malignancy of lung SCC. Further advanced study to understand the pathophysiological functions of podoplanin, including the podoplanin-mediated decreased potential for lymph node metastasis in cancer cells, would provide beneficial information to explore a novel therapeutic strategy for patients with lung SCC.

## Methods

### Cells and Reagents

The lung squamoid cancer cell lines EBC-1 and H157 were maintained with RPMI 1640 (Sigma-Aldrich Japan, Tokyo, Japan) supplemented with 100 units/mL penicillin/streptomycin and 10% fetal bovine serum (FBS). Human fibroblast (MRC5) and mouse fibroblast (NIH3T3) were purchased from the American Type Culture Collection and maintained with Dulbecco's Modified Eagle's Medium supplemented with 10% FBS. Human microvascular endothelial cells (HMVECs) were purchased from Kurabo Co. Ltd., Tokyo, Japan, and maintained with EGM2 medium (Kurabo). c-Jun N-terminal kinase (JNK) inhibitor sp600125 and ROCK/Rho kinase inhibitor Y-27632 were purchased from Promega K.K., Tokyo, Japan. Anti-human podoplanin mouse monoclonal antibody was purchased from AngioBio Co., Del Mar, CA. Anti-total JNK, anti-phospho-JNK, anti-phospho-ERM and anti-total ERM were purchased from Cell Signaling Technology, Beverly, MA. Anti-β-actin rabbit polyclonal antibody was purchased from Sigma-Aldrich Japan, Tokyo, Japan. Anti-murine lymphatic vessel endothelial hyaluronan receptor-1 (LYVE-1) rabbit polyclonal antibody was produced in our laboratory as described previously [38], and anti-mouse CD31 rabbit polyclonal antibody was purchased from Abcam Inc., Cambridge, MA.

### Animals

Male BALB/c nu/nu mice (5 weeks old) were from Kyudo Co., Ltd. (Tosu, Saga, Japan). All animal experiments were done under approved protocols and in accordance with recommendations for the proper care and use of laboratory animals by the Committee for Animal, Recombinant DNA, and Infectious Pathogen Experiments at Kyushu University and according to the Law (No. 105) and Notification (No. 6) of the Japanese Government.

### Reverse transcription polymerase chain reaction (RT-PCR) and real-time RT-PCR

Total cellular RNA was extracted from culture cells or implanted tumor tissues with the ISOGEN system (Wako Pure Chemical Industries, Osaka, Japan) according to the manufacturer's instructions, and treated with RNase-free DNase I (Behringer Roche Applied Science Japan, Tokyo, Japan). Subsequently, aliquots (25 ng) of total RNA were reverse-transcribed and used for PCR templates. Real-time RT-PCR was performed for quantification of gene expression levels. Amplification of target genes was monitored in real-time, and gene expression levels were quantified using the Sequence Detection System, model 7000 (Applied Biosystems Ltd., Tokyo, Japan), according to the manufacturer's instructions for TaqMan methods. The oligonucleotide sequences of PCR primer sets and TaqMan probes were listed in additional file 4. The target gene expression level was normalized with GAPDH level and expressed as relative fold increases compared to mean level in control group.

### Construction of plasmid vector and plasmid templates

The PCR primers incorporating Hind-III and Xho-I sites for amplification of human *podoplanin *are as follows: 5'-GATGTGGAAGGTGTCAGCTC-3'(forward) and 5'-GATCCTCGATGCGAATGCCT-3' (reverse). A PCR amplicon using cDNA from HMVECs was inserted into a plasmid vector, pCEP4, for mammalian cell expression according to general subcloning methods. The primer sequences for construction of plasmid templates are as follows: human and mouse *vegf-c*, 5'-GAAATTACAGTGCCTCTCTC-3' (forward) and 5'-CTAGTTCTTTGTGGGTCCAC-3' (reverse). Each PCR amplicon using cDNA from MRC5 or NIH3T3 was inserted into a plasmid with a pCRII TA cloning kit (Invitrogen, Carlsbad, CA), according to the manufacturer's instructions. The sequence of the insert was examined using the CEQ 2000 Sequence Detection System (Beckman Coulter, Inc., Fullerton, CA), and complete matching was confirmed through comparison to those reported in GenBank (accession nos. AF 390106, NM 005429, and NM 009506 for human *podoplanin*, human *vegf-c*, and mouse *vegf-c*, respectively) was confirmed.

### Establishment of stable transformant

Constructed pCEP-4 inserted human *podoplanin *cDNA and empty pCEP-4 were transfected into EBC-1 cells using LipofectAMINE 2000 (LF2000) reagent (Invitrogen) according to the manufacturer's instructions. Forty-eight hours after transfection, the culture medium was replaced with medium containing 400 μg/mL hygromysin (RPMI-hygro). At that concentration, wild EBC-1 cells were completely killed. The cells were then maintained with RPMI-hygro until the selected cells had grown appropriately. Next, the selected cells were spread onto 96-multiwell plates for single-cell culture and were maintained with RPMI-hygro until they reached confluence. Single-cell-derived confluent cells were continuously maintained in RPMI-hygro in larger shares.

### Tumor implantation model

Under sufficient anesthesia by an i.p. injection of sodium pentobarbital, 1 × 10^6 ^of EBC1-V1, EBC1-V2, EBC1-P4, and EBC1-P15 cells were subcutaneously injected into the dorsal region. After inoculation, tumor length and width were measured every 3 days for 3 weeks, and tumor volume was estimated by the formula *V *= *π/6 *× *a*^*2 *^× *b*, where *a *was the short axis and *b *the long [43]. 21 days after implantation, mice were sacrificed and the primary tumors and axillary lymph nodes (two lymph nodes per mouse) were harvested. Each harvested primary tumor tissue was sliced in two, and the slices were used as samples for histological and molecular biological experiments. Harvested axillary lymph nodes were subjected to HE staining. Through microscopic findings, metastatic status was divided into two cases--positive and negative--irrespective of the area of metastatic foci. The positive lymph nodes were counted, and the incidence of metastasis was expressed as the ratio of positive lymph nodes to the total number of lymph nodes in each group.

### Western blot analysis

Cells were lysed with 200 ml of cell lysis buffer (Promega) containing a cocktail of protease inhibitors (Nacalai Tesque), and the supernatant of the lysed cells was recovered. An aliquot of 20 mg of proteins was subjected to sodium dodecyl sulfate-polyacrylamide gel electrophoresis (SDS-PAGE) under reducing condition, and were then transferred to a PVDF membrane. An hour after being blocked with PBS containing 5% non-fat milk, the membrane was incubated overnight, with each primary antibody diluted with PBS containing 5% BSA and 0.1% Tween 20, at 4°C. The dilution rate was determined according to the manufacturer's instructions. Following several washings with PBS containing 0.1% Tween 20, the membrane was treated for 2 hours with an appropriate HRP-labeled secondary antibody, HistoFine (DAKO, Glostrup, Denmark) at room temperature. The target proteins were visualized by a luminal chemiluminescent reagent, LumiGLO (Cell Signaling Technology). After that, the membrane was subjected to re-probing assay. Briefly, following stripping the binding antibodies using Stripping Solution (Nacalai Tesque, Kyoto Japan) according to the manufacture's instruction, the membrane was washed with PBS and subjected to the WB described above. The band intensity was measured with FMBIO (Hitachi Software Engineering Co. Ltd., Tokyo, Japan).

### *In vitro *proliferation assay

The WST-8 assay kit (Nacalai Tesque) was used to determine the proliferation rates of EBC1-Vs cells and EBC1-Ps cells, which were seeded at 1 × 10^3 ^cells/well on 96-well plates (n = 12 each). Cell viability was evaluated every day for 3 days according to the distributor's protocol. The obtained data were expressed as relative fold increases of O.D. values compared to those 1 day after dissemination.

### Migration assay

Boyden chamber cell invasion was assayed using a cell culture-chamber-insert system (BD, FALCON) with an 8 μm polyethylene terephthalate (PET) membrane. First, 1 × 10^5 ^cells were seeded on the upper chamber in RPMI medium with 1% FBS. The RPMI medium with 1% or 10% FBS was added to the lower chamber. After 18 hours, cells that did not cross the membrane were scraped off the upper side of the membrane with a cotton swab. Cells that had transmigrated to the lower side were fixed with 70% ethanol and subjected to Giemsa staining. The membrane was excised from its support and mounted on a glass slide. Migrating cells in four independent microscopic visual fields (×100) were counted, and expressed as mean number per one field.

### Immunohistochemistry

Angiogenesis and lymphangiogenesis in implanted tumor tissue were immunohistochemically evaluated. Detail immunohistochemcial procedures were described previously [38]. The lymphatic vessels and blood vessels were identified as LYVE-1-positive and CD31-positive vessels, respectively. Image J Software was used to count the total numbers and measure the areas and perimeters of vessels whose apparent luminal areas were framed by LYVE-1-positive or CD31-positive endothelial cells in viable tumors. The blood and lymphatic vessel number was expressed as that per unit of viable tumor area, and the vessel area was expressed as that per a vessel. All sections were independently evaluated by three persons (K.S., M.O., H.S.).

### siRNA transfection

Human podoplanin siRNA was purchased from Invitrogen. The RNA sequence is as below; 5'-UAUAGCGGUCUUCGCUGGUUCCUGG-3'. Control siRNA High GC Duplex (Invitrogen) was used as a negative control. siRNA transfection was performed with lipofectamine RNAiMAX (Invitrogen) according to the manufacturer's instructions.

### Enzyme-linked immunosorbent assay (ELISA)

VEGF-C content in the culture media was measured using Quantikine Immunoassay systems for human VEGF-C (R&D Systems) according to the manufacturer's instructions. Three independent experiments were performed and the obtained data were statistically analyzed. VEGF-C content in the culture media was expressed as the amount of protein secreted from 100,000 cells during 24 hours.

### Statistical analysis

All data were expressed as means ± SD and were analyzed by one-way ANOVA with Fisher's adjustment. Statistical significance was determined using the log-rank test, and *p *< 0.05 was considered statistically significant. Statistical analysis was done using SPSS software version 9.0 (SPSS, Chicago, IL).

## Competing interests

The authors declare that they have no competing interests.

## Authors' contributions

HS carried out experiments, interpreted and analyzed the results and wrote the manuscript. MO designed, interpreted and analyzed the results and wrote the manuscript. YY, SN and KS conceived of the study, and participated in its design and coordination and helped to draft the manuscript. All authors read and approved the final manuscript.

## Supplementary Material

Additional file 1 **Podoplanin-mediated promotion of the migration activity of oral squamoid cancer cells**. Methods, results (graphs) and legends of an in vitro proliferation assay and a migration assay are provided. Click here for file

Additional file 2 **A similar immunohistochemical staining pattern between mouse LYVE-1-positive and mouse podoplanin-positive vessels in an implanted tumor tissue**. Methods, results (photographs) and legends of immunohistochemical studies were shown. Click here for file

Additional file 3 **The podoplanin-mediated reduction of VEGF-C secretion but not of VEGF-A secretion in EBC1 cells *in vitro***. Methods, results (graphs) and legends of Enzyme-linked immunosorbent assay (ELISA) were shown. Click here for file

Additional file 4 **Information of primer sets and TaqMan probes for human specific and mouse specific real-time RT-PCR and semi-quantitative RT-PCR**. A table listing oligonucleotide sequences of PCR primer sets and TaqMan probes, and legends were shown. Click here for file
