# Supplementary material for: Podoplanin in cancer cells is experimentally able to attenuate prolymphangiogenic and lymphogenous metastatic potentials of lung squamoid cancer cells
Source: Mol Cancer. 2010 Oct 31;9:287. doi: 10.1186/1476-4598-9-287 (PMC2987985; doi:10.1186/1476-4598-9-287)
Supplement: Additional file 1 — Podoplanin-mediated promotion of the migration activity of oral squamoid cancer cells. Methods, results (graphs) and legends of an in vitro proliferation assay and a migration assay are provided. [file 1476-4598-9-287-S1.PDF]

Additional file 1

Methods:

Oral squamous cell carcinoma cell line SAS was maintained with RPMI 1640 supplemented with 100 units/mL penicillin/streptomycin and 10% fetal bovine serum (FBS). Constructed pCEP-4 inserted with human podoplanin cDNA or empty pCEP-4 was transfected into SAS cells. Subsequently, stable transformants were established as described in Methods in the main text. Several stable clones were obtained, and SAS-P9 with the highest level of podoplanin among the established clones was adopted for use in the following experiments (data not shown). Control clone SAS-V with an empty vector was randomly adopted. Using these stable clones, an in vitro proliferation assay, migration assay and Western blot analysis were performed as described in Methods in the main text.

Results:

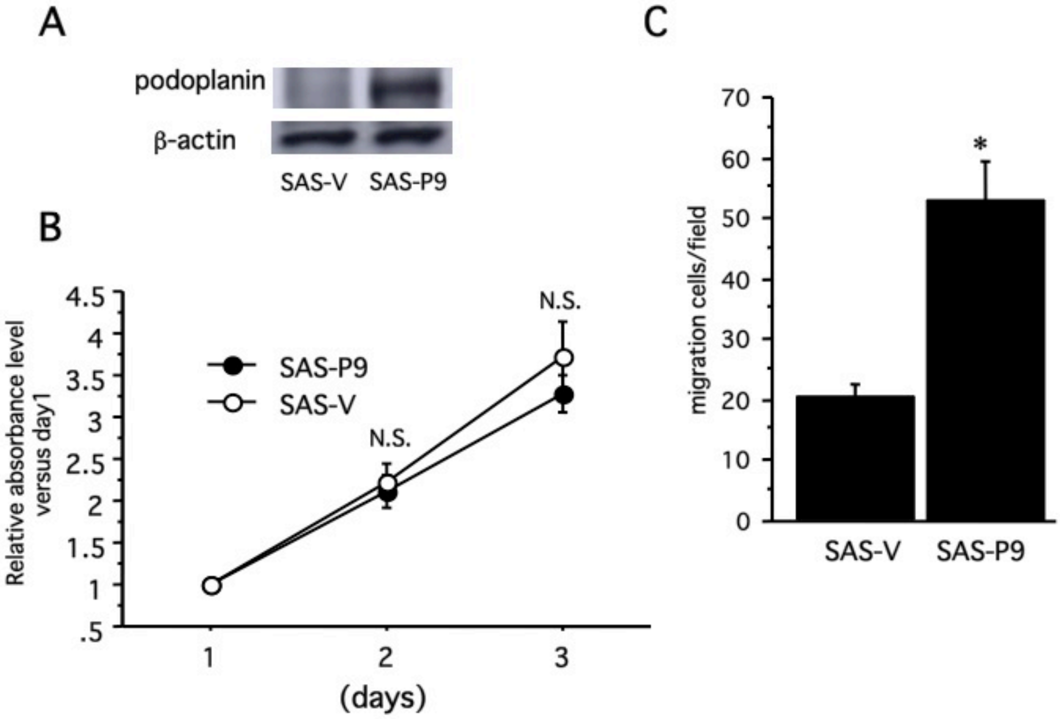

Legends: A) Cell lysates from the cultured stable clones indicated were subjected to Western blot analysis for human podoplanin (upper panel) and subsequently to

re-probing for β-actin (lower panel). Exogenous podoplanin was highly expressed in SAS-P9 cells compared to the endogenous level in SAS-V cells. B) The growth activities of each established clone indicated were examined. An in vitro proliferation assay demonstrated that exogenous podoplanin has no influence on the proliferative activity of SAS cells. NS: no significant change. C) The migration activities of the established clones indicated were examined. A migration assay demonstrated that exogenous podoplanin significantly promotes the migration activity of SAS cells (* $p < 0.005$, $n = 5$ each).
