# Supplementary material for: Podoplanin in cancer cells is experimentally able to attenuate prolymphangiogenic and lymphogenous metastatic potentials of lung squamoid cancer cells
Source: Mol Cancer. 2010 Oct 31;9:287. doi: 10.1186/1476-4598-9-287 (PMC2987985; doi:10.1186/1476-4598-9-287)
Supplement: Additional file 2 — A similar immunohistochemical staining pattern between mouse LYVE-1-positive and mouse podoplanin-positive vessels in an implanted tumor tissue. Methods, results (photographs) and legends of immunohistochemical studies were shown. [file 1476-4598-9-287-S2.PDF]

Additional file 2

Methods: Immunohistochemical stains for mouse LYVE-1 and mouse podoplanin using paraffin-embedded serial sections of EBC1-V1-derived implanted tumor tissue. Anti-mouse podoplanin hamster monoclonal antibody was purchased from AngioBio Co., Del Mar, CA, and used according to the manufacture's instruction. The LYVE-1-positive staining patterns are similar to that of mouse podoplanin.

Results:

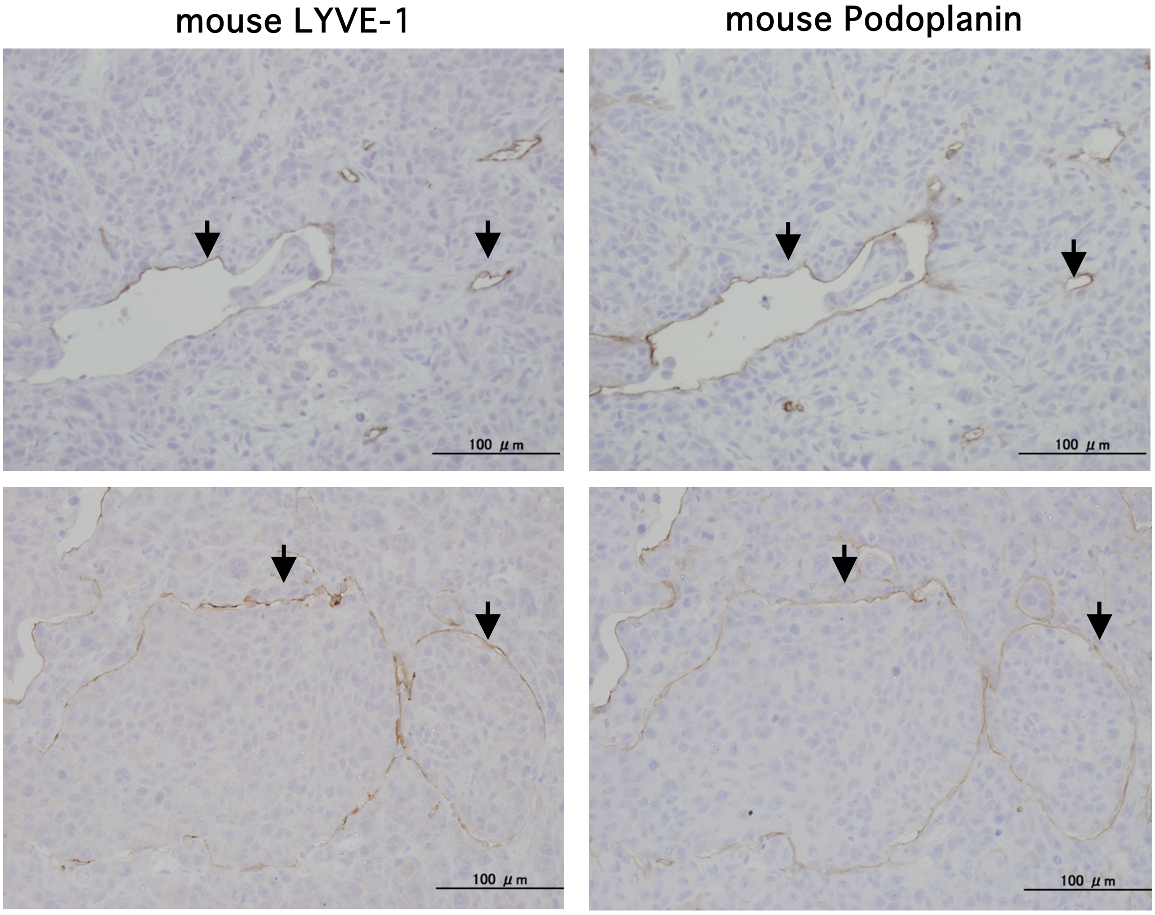

Legends: The two different fields (upper and lower) of immunohistochemical results for mouse LYVE-1 (left photographs) and mouse podoplanin (right photographs) are indicated. A similar staining pattern is observed between LYVE-1 and podoplanin (arrows).
