# Supplementary material for: Podoplanin in cancer cells is experimentally able to attenuate prolymphangiogenic and lymphogenous metastatic potentials of lung squamoid cancer cells
Source: Mol Cancer. 2010 Oct 31;9:287. doi: 10.1186/1476-4598-9-287 (PMC2987985; doi:10.1186/1476-4598-9-287)
Supplement: Additional file 3 — The podoplanin-mediated reduction of VEGF-C secretion but not of VEGF-A secretion in EBC1 cells in vitro. Methods, results (graphs) and legends of Enzyme-linked immunosorbent assay (ELISA) were shown. [file 1476-4598-9-287-S3.PDF]

**Additional file 3**

Methods: VEGF-C, VEGF-A and PDGF-BB contents in the culture medium were determined using Quantikine Immunoassay systems for human VEGF-C, for human VEGF165 and for human PDGF-BB, respectively, according to the manufacturer's instructions (R & D system). For the ELISA, $1\times10^5$ tumor cells were disseminated and grown to sub-confluence in 6-well culture plates, and the media was replaced with serum-deprived medium and cultured. Twenty-four hours later, the media was replaced with fresh medium for 24 hours, and harvested as samples.

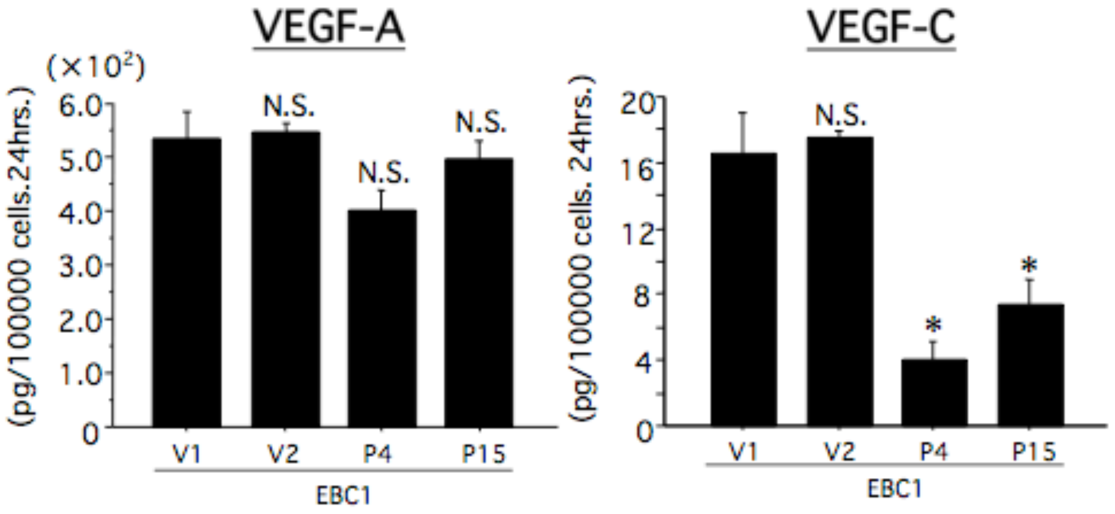


After 24-hour cultivation of EBC1-Ps and EBC1-Vs cells, the harvested media were subjected to ELISA for human VEGF-A or -C. VEGF-A content or VEGF-C content in culture medium was expressed as the amount of protein secreted from $1\times10^5$ cells for 24 hours (hrs.) (* $p < 0.05$, $n = 3$, each group).
