# Supplementary material for: Podoplanin in cancer cells is experimentally able to attenuate prolymphangiogenic and lymphogenous metastatic potentials of lung squamoid cancer cells
Source: Mol Cancer. 2010 Oct 31;9:287. doi: 10.1186/1476-4598-9-287 (PMC2987985; doi:10.1186/1476-4598-9-287)
Supplement: Additional file 4 — Information of primer sets and TaqMan probes for human specific and mouse specific real-time RT-PCR and semi-quantitative RT-PCR. A table listing oligonucleotide sequences of PCR primer sets and TaqMan probes, and legends were shown. [file 1476-4598-9-287-S4.PDF]

Additional file 4

A) Primer sets and TaqMan Probes for human specific real-time RT-PCR

human VEGF-A

TagMan 5’-FAM-actgccatccaatcgagaccctgg-TAMRA-3’

F: 5’-gaagtggtgaagttcatggatgtctat-3’ R: 5’-tcagggtactcctggaagatgtc-3’

human VEGF-C

TagMan 5’-FAM-tgccccaccaattacatgtggaataatcac-TAMRA-3’

F: 5’-tgtcaggcagcgaacaagac-3’ R: 5’-atcttcctgagccaggcatct-3’

human PDGF-B

TagMan 5’-FAM-tggtcagggaacccaggctccttct-TAMRA-3’

F: 5’-gccgagttggacctgaacat -3’ R: 5’-tcttgcactcggcgatcat-3’

human podoplanin

TagMan 5’-FAM-tgacaactctggtggcaacaagtgt-TAMRA-3’

F: 5’-ccagcgaagaccgctataagtc-3’ R: 5’-ctcgatgcgaatgcctgtta-3’

human GAPDH

TagMan 5’-VIC-caagatcatcagcaatgcctcctgca-TAMRA-3’

F: 5’-gtcatgggtgtgaaccatgag-3’ R: 5’-actgtggtcatgagtccttcca-3’

B) Primer sets and TaqMan Probes for mouse specific real-time RT-PCR

mouse VEGF-C

TagMan 5’-FAM- tgccagcaacattaccacagtgtcagg-TAMRA-3’

F: 5’- ccggtgcatgtctaaactggat-3’ R: 5’- gcatcggcacatgtagttatt-3’

C) Primer sets for human specific RT-PCR

human Podoplanin (AT:60, CN:35)

F: 5’-gatgtggaaggtgtcagctc-3’ R: 5’-gatcctcgatgcgaatgcct-3’

human HGF (AT:60, CN:30)

F: 5’-ttacgagtggcacatctctata-3’ R: 5’-tgactgtggtaccttatatgtta-3’

human VEGF-D (AT:60, CN:30)

F: 5’-gtggctgttgcaatgaagaga-3’ R: 5’-tgcactcaaagcaactgcagtt-3’

human Ang-2 (AT:60, CN:30)

F: 5’-agatcaaggcctactgtgacat-3’ R: 5’-caaatacatttgtcgttgtctcc-3’

human GAPDH (AT:60, CN:18)

F: 5’-accagggctgcttttaactc-3’ R: 5’-tcgccccacttgattttgga-3’

Oligonucleotide sequences of TaqMan probes and primer sets for real-time RT-PCR (A and B) and primer sets for semi-quantitative RT-PCR (C) were listed. All primer sets were designed specifically to react to the species of targets. F, forward primer; R, reverse primer; AT, annealing temperature; CN, cycle number.
